# Storm water contamination and its effect on the quality of urban surface waters

**DOI:** 10.1007/s10661-014-3889-0

**Published:** 2014-07-02

**Authors:** Danuta Barałkiewicz, Maria Chudzińska, Barbara Szpakowska, Dariusz Świerk, Ryszard Gołdyn, Renata Dondajewska

**Affiliations:** 1 Faculty of Chemistry, Department of Trace Elements Analysis by Spectroscopy Method, Adam Mickiewicz University, Umultowska 89b, 61-614 Poznań, Poland; 2 Faculty of Horticulture and Landscape Architecture, Department of Landscape Architecture, Poznań University of Life Sciences, Dąbrowskiego 159, 60-594 Poznań, Poland; 3 Faculty of Biology, Department of Water Protection, Adam Mickiewicz University, Umultowska 89, 61-614 Poznań, Poland

**Keywords:** Rainwater, Surface waters, Storm water, Land use, Pollutants, Biota, Urban area

## Abstract

We studied the effect of storm water drained by the sewerage system and discharged into a river and a small reservoir, on the example of five catchments located within the boundaries of the city of Poznań (Poland). These catchments differed both in terms of their surface area and land use (single- and multi-family housing, industrial areas). The aim of the analyses was to explain to what extent pollutants found in storm water runoff from the studied catchments affected the quality of surface waters and whether it threatened the aquatic organisms. Only some of the 14 studied variables and 22 chemical elements were important for the water quality of the river, i.e., pH, TSS, rain intensity, temperature, conductivity, dissolved oxygen, organic matter content, Al, Cu, Pb, Zn, Fe, Cd, Ni, Se, and Tl. The most serious threat to biota in the receiver came from the copper contamination of storm water runoff. Of all samples below the sewerage outflow, 74 % exceeded the mean acute value for *Daphnia* species. Some of them exceeded safe concentrations for other aquatic organisms. Only the outlet from the industrial area with the highest impervious surface had a substantial influence on the water quality of the river. A reservoir situated in the river course had an important influence on the elimination of storm water pollution, despite the very short residence time of its water.

## Introduction

Rainwater quality is affected by the type of street paving and of the catchment, the season of the year, and the intensity of vehicle traffic (Shirasuna et al. 2006). Rainwater may also contain fuel combustion products emitted to the atmosphere, components originating from industrial emissions, mineral particles from the ground surface, etc. (Walna and Kurzyca 2009; Ki et al. 2011). The quality of the rainwater may depend on the form in which pollutants are found (Dougherty et al. 2006). The more strongly specific pollutants are bound with solid particles, the greater is the effect of heavy but short-term rain on the load of flushed pollutants (Budai and Clement 2007; Brodie and Egodawalta 2011). Precipitation waters discharged through the storm water drainage system not only influence physicochemical variables of the receiving waters but also have an impact on organisms living in the water column and in the bottom sediments (Marsalek et al. 1999; Gołdyn and Szeląg-Wasielewska 2005; Johnson et al. 2011; Rossi et al. 2013).

For many years, urban rainwaters discharged through the storm water sewerage system into the receiver were considered as clean in Poland waters. Thus, the quality of storm water was rarely analyzed, and, when it was, only some of the water quality indices were measured, such as pH, total suspended solids (TSS), biochemical oxygen demand (BOD_5_), chemical oxygen demand (COD), chlorides, electrolytic conductivity, or sulfates (Kasterka and Kasterka 1998; Sawicka-Siarkiewicz 2006). Only in recent years has rainwater runoff been included in the category of wastewater, which required it to be treated before its discharge to surface waters, if standard limits for TSS and PAHs were exceeded (Ministry of Environment Regulation 2006). The problem of rainwater has not been properly regulated by the EU laws to date. The Sewage Directive (Council Directive 1991), repeatedly amended over the years, only limits permissible pollution caused by storm water overflows discharging to the receiver situated on combined sewers. The main EU legal act, i.e., the Water Framework Directive (2000), does not recognize storm water runoff as an important cause of water pollution (in rivers and lakes). Perhaps, this is the reason why in Europe (compared to the USA), so little is known of the impact of rainwater runoff on the receiver water quality and its toxicity to aquatic organisms. Under the US law, the Clean Water Act (1972) requires the EPA to publish and periodically update ambient water quality criteria (Burton and Pitt 2001). The first criteria for acceptable concentrations of the pollutants were published in 1976 (EPA 1976). These criteria have been revised many times since (EPA 2009). They refer to the “criterion of maximum concentration” (CMC) with an exposure period of 1 h (acute criterion) and to the “criterion of continuous concentration” (CCC) a 4-day period (chronic criterion). The idea of these criteria is to protect the organisms living in waters of the receivers against the pollutants discharged from various outlets.

The aim of the presented studies was to determine the effect of storm water outflow from small catchments of different land use (single- and multi-family housing, industrial areas) on the quality of surface waters (the Cybina River and the Antoninek Reservoir) in the city of Poznań. In particular, we were interested in how the type of catchment influences the concentrations of various pollutants in storm water runoff and whether it poses a risk for the aquatic biota.

## Materials and methods

### Study area

The research took place in 2009 along the lower section of the Cybina River, which is the right tributary of the Warta River. The analyzed section of the river is located in the city of Poznań, between Lake Swarzędzkie and Lake Maltańskie, i.e., between 4.3 and 8.7 km of the river course, counting from its confluence with the Warta River. Mean multi-annual water flow rate in this section of the river was 0.67 m^3^ s^−1^. In the 1980s, four small dammed reservoirs were created or reconstructed in this section to intensify self-purification of the waters (Gołdyn et al. 2009).

**Fig. 1:**
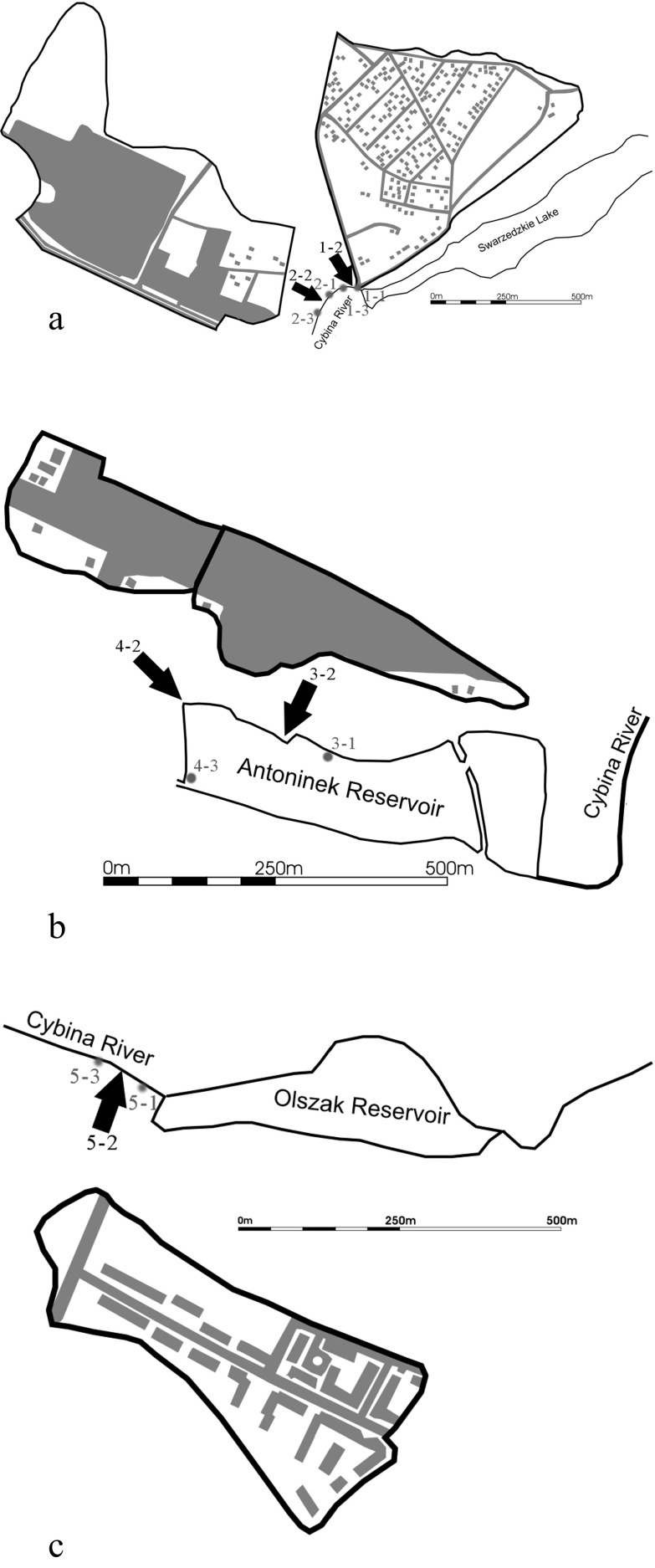
The location map for water sampling stations. **a** below Lake Swarzędzkie, **b** from the Antoninek Reservoir, and **c** below the Olszak Reservoir

The effect of storm water runoff on the surface waters was studied on the basis of five catchments in which storm water entered the drainage system and then discharged to the Cybina and to a small rheolimnic reservoir (mean residence time of water −0.5 day) located on this river—the Antoninek Reservoir. Catchment no. 1 responsible for draining an area with single-family housing (residential area) covers an area of 42.7 ha, of which 28.9 % of the surface is impervious (Fig. 1a). A sedimentation tank and a separator are located in front of the outlet of the sewer to the Cybina to provide a storm water pretreatment. Catchment no. 2 covers the area of a car-producing plant with parking lots for newly produced vehicles and for cars used by the plant employees, as well as a section of motorway of approximately 800 m in length. The drained catchment is 56.8 ha in size, of which 51.6 % is impervious (Fig. 1a). Before being discharged to the Cybina River, storm water is pretreated in an Imhoff sedimentation tank, adapted for this purpose from a former treatment plant processing wastewater and household sewage. Catchment no. 3 mainly covers the area of a glass factory. It has an area of 4.78 ha, of which 88 % is impervious (Fig. 1b). Storm water is discharged to the Antoninek Reservoir. Catchment no. 4 covers an area of workshops, a showroom, and the parking lots of a car dealer and servicing company. The catchment is 4.33 ha large, of which 76 % is impervious. Storm water is discharged to the Antoninek Reservoir (Fig. 1b). Catchment no. 5 covers the area of a small multi-family housing district (with apartment buildings). The catchment is 7.37 ha large, of which only 26.7 % is impervious (Fig. 1c). The small impervious area and a slight slope result in lower amounts of runoff in comparison with those of the other catchments. The runoff is discharged directly to the Cybina River.

The studied catchments are located on outwash deposits, associated with the Poznan phase of Baltic glaciation. On the surface, however, they are often covered with soil overlays of human origin. The rainwater sewer system is made of concrete pipes of different diameters, from 20 cm to 1.2 m.

The largest impervious area, resulting in the highest amount of storm waters discharged to the Cybina River, is in catchment no. 2 (29.3 ha); catchment no. 1 covers a smaller area (12.3 ha), while the impervious area in the rest of the studied catchments (nos. 3–5) is many times smaller: 4.2, 3.3, and 2.0 ha, respectively. During strong rain events, waterflow below sewer no. 2, responsible for draining the area of the car-producing plant (Fig. 1a), and rose twofold, while the difference was not visible for water below sewers 3 to 5.

During sampling, rainfall intensity was estimated using a 4-point scale: 1—rain with a very low intensity (drizzle), usually below 0.5 mm h^−1^; 2—average rain of frontal type, between 0.5 and 2 mm h^−1^; 3—heavy rain of convective type, between 2 and 4 mm h^−1^; and 4—storm rainfall, over 4 mm h^−1^.

### Sample collection

A total of 13 sampling stations were established. Five of them were outlets of rainwater sewers (marked with arrows), and eight were located on the receivers (river and water reservoir). The stations on the river were located above and below the storm water outlets; however, in the case of the reservoir, due to the storm water outlets being located close to each other, only one station was established above and one below both outlets (Fig. 1b).

Water samples from the river were collected together with the collection of storm water samples. Water and storm water were sampled once or twice per month during the year, with the exception of August, depending on the rain frequency. Samples were collected from the thalweg of a stream using a grab sampler. Storm water was sampled directly from the outflows of the sewers. Samples were collected for chemical analyses from each station in three replications and they were assayed separately. Immediately after collection, samples were delivered to the laboratory and analyzed on the same day. In case of element analysis, samples were acidified with nitric acid.

### Analytical methods

We analyzed 14 basic physicochemical variables in water and storm water, i.e., dissolved phosphates and total phosphorus; ammonium, nitrate, nitrite, organic, and total nitrogen; temperature; pH; dissolved oxygen and its saturation; BOD_5_; seston with TSS and conductivity. Temperature, pH, conductivity, and dissolved oxygen and its saturation were measured in situ using a WTW Multi 350i-meter. Other variables were analyzed according to the European Standards, i.e., dissolved reactive phosphorus (DRP) and total phosphorus (TP) were analyzed spectrophotometrically using the molybdate method with ascorbic acid as a reducer (TP after the persulfate digestion procedure), ammonium nitrogen using the Nessler method, nitrate N using the salicylate method, nitrite N using the diazotization method with sulfanilic acid, organic nitrogen using the Kjeldahl digestion method, BOD_5_ using the Winkler method (Elbanowska et al. 1999), and total suspended solids using the gravimetric method with filtration through Whatman glass fiber filters GF/C (Wetzel and Likens 2000). Twenty-two elements (Ag, Al, As, Ba, Ca, Cd, Co, Cr, Cu, Fe, K, Li, Mg, Mn, Na, Ni, Pb, Se, Sr, Tl, V, and Zn) were analyzed in each water sample. The use of flame atomic absorption spectrometry or flame atomic emission spectrometry techniques, F-AAS/F-AES according to the ISO standards (ISO 9964-1 1993; ISO 7980 1986) and inductively coupled plasma mass spectrometry technique, ICP-MS using ISO 17294-2 (2003) standard made it possible to determine these elements with high precision and accuracy. A standard ELAN DRC II (Perkin Elmer Sciex, Canada) ICP-MS spectrometer equipped with a Meinhard concentric nebulizer, cyclonic spray chamber, Pt cones, and quadruple mass analyzer was used for this study. Typical instrument operating conditions for the ICP-MS spectrometer were as follows: RF power—1,200 W, plasma Ar flow rate—15 L/min, nebulizer Ar flow rate—0.94 L/min, and auxiliary Ar flow rate—1.2 L/min. The isotopes of Sc^45^, Y^89^, and Tb^159^ were applied as internal standards in order to effectively correct for temporal variations in signal intensity.

For the major elements (Ca, Fe, K, Mg, Na, and Zn), F-AAS/AES techniques were employed, according to the following settings: the wavelengths were 422.7, 248.3, 766.5, 202.6, 589.0, and 213.9 nm appropriately and the widths of split were 0.5 nm (Ca), 1.0 nm (Mg, Zn), and 0.2 nm for the rest of the elements.

Prior to the analyses of unknown samples, the ICP-MS, F-AAS, and F-AES methods were validated by analyzing the water reference material NIST 1643e. In the course of the study, the control material was run every 10 samples to ensure analytical accuracy. The QC data are given in Table 1.

**Table 1:** Comparison of certified mass concentration and determination value (in μg L^−1^)

Element	Certified value	Obtained value
Al	142.0 ± 3.5	141.8 ± 0.8
As	60.5 ± 0.73	56.3 ± 1.5
Ba	544.0 ± 8.9	534.7 ± 15.3
Ca	(32.3 ± 0.5) · 10^3^	(31.4 ± 2.6) · 10^3^
Cd	6.57 ± 0.37	6.18 ± 3.2
Co	27.6 ± 0.3	25.8 ± 0.4
Cr	20.4 ± 0.2	21.3 ± 3.2
Cu	22.8 ± 3.8	21.9 ± 3.0
K	(2.03 ± 0.22) · 10^3^	(1.81 ± 0.28) · 10^3^
Mg	(8.04 ± 0.04) · 10^3^	(8.15 ± 1.0) · 10^3^
Mn	39.00 ± 0.83	36.23 ± 2.21
Na	(20.7 ± 0.6) · 10^3^	(22.1 ± 4.1) · 10^3^
Ni	62.4 ± 2.7	60.3 ± 1.0
Pb	19.6 ± 0.6	18.9 ± 3.1
Se	58.3 ± 0.37	61.8 ± 0.27
Sr	320.0 ± 7.3	316.9 ± 9.3
Zn	78.5 ± 0.6	75.1 ± 3.1

### Statistical analysis

Correlation analysis showed that the length of the gradient (2.6) did not exceed the value of 3, which made it possible to apply a linear model. Statistical analysis (Canoco 4.5 for Windows) was conducted applying the canonical variate analysis (CVA), which is a canonical variation of linear discriminant analysis. This analysis indicates which explanatory variables are the best predictors of sampling points (storm water, river upstream and downstream), taking into account types of catchment area and rainfall intensity during sampling. Using this analysis, three models were constructed, predicting trace elements, macroelements together with forms of nitrogen and phosphorus, and environmental variables (temperature, oxygen content, BOD_5_, electrolytic conductivity, pH, TSS). In order to determine the boundary level of significance, the Monte Carlo permutation test was used. Calculations were presented graphically in the CanoDraw for Windows program.

Differences between the stations were tested with the test of matched groups and the non-parametric Mann-Whitney *U* test, using STATISTICA version 6.0 software.

## Results and discussion

### Spatial variability

The temperature of the storm water changed with the seasons. It was higher by approximately 1 °C during winter and lower by 1–4 °C during summer, compared to the water in the Cybina River and in the Antoninek Reservoir. The impact of storm water on the water temperature of the receiver was small. The greatest single decrease of 2.6 °C was found for the largest storm water runoff (station 2), but a statistically significant change was stated only at station 1 (Table 2).

**Table 2:** Increase (positive values) or decrease (negative values) of the mean values of the studied variables and the elements in Cybina River (stations 1, 2, and 5) and in Antoninek Reservoir (stations 3 and 4) under the influence of storm water discharged by sewer nos. 1–5, i.e., the differences between the values above and below the storm water discharge and percentage of changes

Variables and elements		Station 1		Station 2		Stations 3 and 4		Station 5	
	Unit	Value	Percent	Value	Percent	Value	Percent	Value	Percent
Temperature	°C	*−0.2*	*−1.6*	−0.4	−2.6	0.2	1.2	0.3	2.0
pH		*−0.16*	*−2.2*	*−0.41*	*−5.6*	0.31	4.3	−0.09	−1.3
Conductivity	μS cm^−1^	−19.7	−2.8	−121.3	−17.1	−20.2	−2.9	*−8.6*	*−1.2*
Oxygen	mg L^−1^ O_2_	0.02	0.4	*0.78*	*13.2*	0.27	3.8	0.28	3.7
Ammonium N	mg L^−1^ N	0.065	5.6	−0.090	−7.6	−0.049	−4.8	0.098	12.2
Nitrite N	mg L^−1^ N	*0.002*	*38.0*	0.002	30.7	−0.001	−15.7	0.000	−9.1
Nitrate N	mg L^−1^ N	0.102	19.0	−0.121	−20.2	0.041	6.7	0.027	4.8
Organic N	mg L^−1^ N	−0.254	−14.5	*−0.587*	*−33.5*	−0.025	−1.6	0.143	0.3
Total N	mg L^−1^ N	−0.006	−0.2	*−0.804*	*−22.6*	−0.092	−2.8	0.223	7.7
DRP	mg L^−1^ P	*0.017*	*9.8*	*−0.073*	*−39.0*	−0.001	−0.6	−0.008	−5.2
TP	mg L^−1^ P	*0.023*	*10.6*	*−0.070*	*−30.4*	0.005	3.0	−0.022	−10.0
TSS	mg L^−1^	*5.21*	*99.4*	*5.90*	*94.7*	−0.85	−5.8	6.10	47.2
BOD_5_	mg L^−1^ O_2_	−0.16	−3.4	0.08	1.8	−1.56	−22.5	1.15	25.5
Li	μg L^−1^	−0.311	−5.6	*−1.259*	*−24.3*	−0.779	−12.8	2.074	33.1
Al	μg L^−1^	23.054	278.9	*23.428*	*119.2*	−7.453	−26.3	7.726	43.6
V	μg L^−1^	*0.184*	*43.3*	*0.206*	*44.3*	−0.259	−28.1	0.068	8.2
Cr	μg L^−1^	0.087	5.3	0.166	8.8	1.044	123.5	−0.142	−4.0
Mn	μg L^−1^	0.709	0.9	−12.364	−15.4	*−15.427*	*−18.8*	2.766	2.8
Co	μg L^−1^	*0.041*	*88.0*	*0.138*	*254.0*	−0.037	−25.5	−0.005	−4.4
Ni	μg L^−1^	−0.015	−1.0	0.256	16.7	−0.577	−22.6	−0.115	−4.8
Cu	μg L^−1^	0.720	18.0	*5.125*	*176.6*	1.326	60.1	4.422	114.6
Zn	μg L^−1^	−3.869	−21.4	*79.335*	*351.5*	−2.437	−9.0	1.045	13.9
As	μg L^−1^	−0.136	−9.0	*−0.415*	*−28.1*	−0.203	−12.6	0.295	15.4
Se	μg L^−1^	−0.035	−34.8	0.000	0.0	0.000	0.0	0.092	128.3
Sr	μg L^−1^	−30.573	−10.2	*−87.655*	*−30.7*	−6.618	−2.2	82.023	22.6
Ag	μg L^−1^	0.002	131.2	0.004	655.8	−0.001	−18.1	0.001	21.1
Cd	μg L^−1^	−0.002	−7.6	*0.055*	*352.9*	*−0.172*	*−86.9*	*0.013*	*159.1*
Ba	μg L^−1^	−6.445	−11.5	−8.438	−16.4	*−28.691*	*−37.5*	−1.670	−2.2
Tl	μg L^−1^	0.001	40.2	*0.003*	*180.9*	−0.006	−63.4	*0.002*	*174.5*
Pb	μg L^−1^	0.244	35.1	*1.595*	*276.9*	−2.485	−32.6	0.446	32.5
Na	mg L^−1^	0.22	0.2	−8.02	−8.9	5.36	6.2	−2.22	−2.4
K	mg L^−1^	*−1.27*	*−4.9*	*−7.53*	*−30.9*	0.64	2.7	*−1.51*	*−5.9*
Mg	mg L^−1^	−0.87	−4.3	*−5.42*	*−25.9*	1.80	10.9	−1.05	−5.3
Ca	mg L^−1^	−0.77	−1.8	*−15.97*	*−35.2*	5.26	13.5	−0.12	−0.2
Fe	mg L^−1^	−0.008	−3.3	0.060	18.4	0.009	2.5	−0.112	−22.7

Statistically significant changes are italicized (*N* = 15)

The pH value of discharged water was very often much lower than that of waters in the river and in the reservoir (by approximately half a unit). Minimal values of pH, which are found on the list of non-priority pollutants (EPA 2009), were lower than 6.5 in storm water discharged by sewer nos. 2 and 5 (Table 3). Such values can have a chronic effect on aquatic organisms. The pH in the river water tended to decrease under the influence of discharged storm water (Table 2).

**Table 3:** Minimum, maximum, and mean values as well as standard deviations for analyzed physicochemical variables in storm waters discharged from sewer nos. 1–5

Variables	1				2				3				4				5			
	Min	Max	$\overline{x}$	SD	Min	Max	$\overline{x}$	SD	Min	Max	$\overline{x}$	SD	Min	Max	$\overline{x}$	SD	Min	Max	$\overline{x}$	SD
Temperature (°C)	2.10	23.30	14.54	6.21	2.10	22.80	14.03	5.78	0.80	23.40	13.50	6.71	1.50	22.90	13.50	6.63	4.50	28.50	14.49	7.68
pH	6.80	8.26			5.96	7.96			6.83	8.38			6.99	8.66			6.04	8.66		
Conductivity (μS cm^−1^)	3.00	1965.00	648.70	357.18	33.00	1418.00	492.20	352.78	65.00	803.00	564.32	194.72	38.00	2406.00	443.45	276.77	296.00	1171.00	700.34	151.72
Oxygen (mg L^−1^ O_2_)	2.00	11.65	4.94	2.68	1.70	11.70	5.98	2.50	4.10	12.37	6.51	2.01	4.60	11.69	6.80	1.90	4.40	11.45	7.16	2.05
Ammonium N (mg L^−1^ N)	0.64	3.37	1.31	0.80	0.54	2.60	1.17	0.58	0.64	2.19	0.92	0.36	0.62	2.19	1.11	0.47	0.38	7.36	1.21	1.32
Nitrite N (mg L^−1^ N)	0.00	0.03	0.01	0.01	0.00	0.03	0.01	0.01	0.00	0.03	0.01	0.01	0.00	0.02	0.01	0.01	0.00	0.04	0.01	0.01
Nitrate N (mg L^−1^ N)	0.00	2.25	0.44	0.74	0.00	1.93	0.49	0.68	0.00	3.02	0.67	0.99	0.00	1.81	0.44	0.63	0.00	4.11	0.90	1.11
Organic N (mg L^−1^ N)	0.19	2.63	1.53	0.70	0.20	3.68	1.36	0.74	0.12	3.94	1.39	0.93	0.07	3.14	1.24	0.92	0.44	5.54	1.71	1.10
Total N (mg L^−1^ N)	1.34	5.77	3.36	1.26	1.27	5.68	3.14	1.18	0.97	5.12	2.94	1.39	0.69	4.33	2.69	1.09	1.08	14.6	3.58	2.60
DRP (mg L^−1^)	0.05	0.43	0.17	0.12	0.00	0.47	0.11	0.12	0.00	0.24	0.07	0.07	0.00	0.22	0.07	0.06	0.03	0.51	0.17	0.13
TP (mg L^−1^)	0.09	0.54	0.22	0.13	0.04	0.52	0.15	0.12	0.02	0.30	0.12	0.08	0.04	0.33	0.12	0.08	0.03	0.57	0.22	0.14
TSS (mg L^−1^)	1.80	344.00	31.76	61.92	2.40	102.00	16.85	17.16	4.00	107.33	23.51	22.05	4.17	736.00	55.31	129.31	3.00	341.00	27.89	49.56
BOD_5_ (mg L^−1^)	1.22	16.84	5.93	3.94	1.37	14.00	4.69	3.07	1.17	15.11	6.55	3.62	2.05	25.11	7.48	4.98	1.27	16.46	5.70	3.63

Electrolytic conductivity in storm water changed over a very wide range of values from 3 to 2,406 μS cm^−1^ (Table 3). High values in runoff found during the winter were caused by snow-melting agents applied to remove snow and prevent its freezing on the roads. They caused an observable increase in conductivity of the water in the receiver (by a maximum of 300 μS cm^−1^ in a single case). However, during other seasons, storm water runoffs were of much lower conductivity than the river water, so their discharge decreased this variable in the receiver (Table 2).

The content of oxygen dissolved in storm water effluents ranged from 1.7 to 12.4 mg L^−1^ O_2_ (Table 3). The lowest values were observed at the outlet of the second rainwater sewer, equipped with the Imhoff sedimentation tank. At the outlet of sewer 1, also equipped with a sedimentation tank, the recorded oxygen content was below 3 mg L^−1^, which could influence the juvenile stages of fish (Brylińska 1986). In most cases, however, storm waters were better oxygenated than the river water. Generally, storm water runoff caused a slight increase in the concentration of oxygen both in the river and in the reservoir (Table 2). However, due to elevated BOD and suspended solids in discharged storm waters, a subsequent decrease in oxygen concentrations is expected as a result of the microbial decomposition of organic matter, as reported in an earlier study (Gołdyn 2000).

Among the mineral forms of nitrogen, the highest values were recorded for ammonium ions. Their content in discharged effluent ranged from 0.38 to 7.36 mg L^−1^ N. Maximum values exceeded the CMC for aquatic organisms in samples taken from sewer no. 5 (EPA 2009). The most frequently recorded values fell within a range from 0.6 to 1.5 mg L^−1^ N. Their impact on the river and the reservoir was not statistically significant because of the simultaneous presence of high concentrations of ammonium nitrogen. The values of nitrite nitrogen were very low, ranging from 0.001 to 0.041 mg L^−1^ N. Nitrate nitrogen concentration in many rainwater runoff samples was below the detection limit. The maximum determined value was 4.11 mg L^−1^ N, but in the receiver, the concentrations were also usually high; thus, the influence of storm water was not statistically significant. Organic nitrogen fell within the range of 0.07–5.54 mg L^−1^ N and was usually lower than in the water of the receiver. A statistically significant decrease of its concentration in the river was observed only in the case of the largest sewer (station 2). Mean values of total nitrogen were above 2 mg L^−1^ N, with a maximum value exceeding 14 mg L^−1^ N, so they could have caused eutrophication in the receiver, particularly when the lower concentrations of nitrogen were present in the river water. Typically, however, total nitrogen concentration in storm water runoff was lower than that in the river causing a decrease of nitrogen concentration in the receiver, which was statistically significant only in the case of station 2 (Table 2).

Levels of phosphate and total phosphorus in storm water runoff have a clear and statistically significant impact on river water. However, for sewer no. 1, a slight increase in concentration in the river was stated, while in the case of sewer 2, a marked decrease in the concentration in the receiver water was found.

TSS content in storm water runoff was usually very high, reaching up to 344 mg L^−1^ (Table 3). This resulted in a clear increase of TSS content in the river water, exceeding 90 % as a mean in the case of sewers 1 and 2 (Table 2). This can affect aquatic organisms as it reduces light penetration in the water, thus limiting primary production. EPA (1986) recommended that the depth of light penetration should not be reduced by more than 10 %. Offsetting the benefit of suspended solids in water is the sorption of organic materials such as pesticides and other pollutants like heavy metals. Sedimentation may remove these materials from the water column (Nocoń et al. 2013; Rossi et al. 2013; Zębek and Szwejkowska 2014). The amounts of heavy metals adsorbed on suspended solids sinking to the bottom sediments of rivers and reservoirs that are storm water receivers can be particularly high and thereby cause a serious threat to the fauna of benthic invertebrates and fish spawning (Rossi et al. 2013). The direct effect of TSS on fish was probably negligible, since only once was the limit value for the protection of fish against exposure to excessive TSS (25 mg L^−1^) exceeded in the river water (Robertson et al. 2006).

BOD_5_ was occasionally high in storm water, reaching up to 25.11 mg L^−1^ O_2_ (Table 3), but only once did it not meet the requirements of the Council Directive 91/271/EEC of 21 May (1991) concerning discharges from urban wastewater treatment plants. However, 45 % of data exceeded 6 mg L^−1^ O_2_, i.e., the limit value for the good ecological status of rivers (Ministry of Environment Regulation 2011). In many cases, BOD_5_ in storm water runoff was lower than that in the river water; hence, the impact on the water quality of the receiver was not statistically significant (Table 2).

According to the EPA (2009), nine of the analyzed elements were priority pollutants. Some of them occurred in the storm water runoff at low concentrations, below the limit of acute and chronic values for aquatic organisms (i.e., chromium, nickel, arsenic, selenium, and silver).

Cadmium in some cases exceeded the limit of chronic concentrations in rainwater runoff, resulting in a statistically significant increase of the concentration in the river, although these concentrations below the discharge, next to mixing with river water, never reached chronic values. The highest concentrations of cadmium were present in the outflow from the area of the glass factory, discharged into Antoninek Reservoir. However, they did not result in an increase of concentrations of this element in the reservoir. On the contrary, a marked reduction of cadmium occurred in the reservoir, which proves the importance of the process of TSS sedimentation together with the adsorbed heavy metals (Rossi et al. 2013). This results in a strong heavy metal contamination of bottom sediments and biota in the reservoirs receiving storm water discharge (Rzętała et al. 2013; Rzymski et al. 2014).

Maximum lead concentrations in the storm water discharged by sewer nos. 3–5 exceeded the limit of acute toxicity to aquatic organisms, i.e., 65 μg L^−1^ (EPA 2009). However, lead was recorded in much lower concentrations in the river water, even downstream of the outlet of the sewers (up to 31.7 μg L^−1^). Only in the case of station 2 was a statistically significant increase in the concentration of lead found in the river (Table 2). The chronic value in water below the sewers was exceeded more frequently, i.e., in 38 % of the samples. This means that in over one third of the rain events, a negative impact of lead concentrations on aquatic biota could have been expected.

Maximum values of zinc concentrations in all sewers exceeded the acute toxicity level, but only in the case of sewer no. 2 did we observe a statistically significant increase in its concentrations in the river. Lethal concentrations of zinc ranged over more than three orders of magnitude from 166 to >67,000 μg L^−1^ (Brinkman and Johnston 2012). Typically, vertebrates are more resistant to heavy metals than invertebrates, especially planktonic cladocerans (Clearwater et al. 2013; Taweel et al. 2013); however, among species native to the Rocky Mountains, trout fish were the most sensitive, while several benthic invertebrates were the most tolerant (Brinkman and Johnston 2012). The source of Zn and Pb in urban areas in Poland is mainly low-chimney emission, due to numerous households heated with coal-fired heating stations (Lis and Pasieczna 2005).

The most serious threat to biota in the receiver came from the copper contamination of storm water runoff. Most of the data from all the sewers exceeded the species mean acute value (SMAV), which for the most sensitive species is 2.37 μg L^−1^ (EPA 2007). These species belong to the common water organism group Cladocera, e.g., *Daphnia pulicaria*, *Daphnia magna*, and *Ceriodaphnia dubia*. Fish are less sensitive; however, SMAV for northern pike at the level of 60.4 μg L^−1^ (EPA l.c.) was frequently exceeded in storm water runoff from sewer nos. 2 and 3. Fortunately, the highest concentration of copper in the river water below the sewer outlets was 46.2 μg L^−1^, so it was not toxic for fish but still very harmful for cladocerans. As many as 74 % of all the samples from the river below the sewer outflows exceeded SMAV for *Daphnia* species.

Aluminum and iron, which according to EPA (2009) belong to the non-priority pollutants, were present in higher concentrations in discharged storm water. Nevertheless, in the river water, only chronic concentrations were observed. Manganese, in turn, exceeded 50 μg L^−1^ both in rainwater discharged from the sewers and in the river water below the sewers, i.e., the safe value for human health related to water consumption (Table 4).

**Table 4:** Minimum, maximum, and mean values as well as standard deviations for analyzed chemical elements in storm waters discharged from sewer nos. 1–5

Element	1				2				3				4				5			
	Min	Max	$\overline{x}$	SD	Min	Max	$\overline{x}$	SD	Min	Max	$\overline{x}$	SD	Min	Max	$\overline{x}$	SD	Min	Max	$\overline{x}$	SD
Li (μg L^−1^)	0.96	13.30	4.77	2.95	0.42	9.15	3.53	2.66	1.98	12.60	5.05	2.92	0.34	8.65	3.95	2.54	2.41	17.20	6.87	3.39
Al (μg L^−1^)	0.00	3490.00	131.31	635.02	0.00	632.00	39.42	117.18	0.00	823.00	47.47	174.75	0.00	1070.00	97.80	300.09	0.00	112.00	13.92	25.75
V (μg L^−1^)	0.05	9.72	1.42	2.12	0.07	2.73	0.92	0.73	0.23	3.28	1.27	0.90	0.01	6.97	1.53	1.57	0.00	8.50	1.54	1.61
Cr (μg L^−1^)	0.00	5.42	1.06	1.20	0.04	6.03	1.17	1.41	0.10	6.40	1.53	1.62	0.17	4.90	1.43	1.44	0.15	12.70	1.69	2.48
Mn (μg L^−1^)	20.30	306.00	114.36	93.11	8.04	261.00	63.55	66.58	8.10	227.00	79.20	56.65	7.72	207.00	59.31	48.88	21.00	288.00	117.53	79.91
Co (μg L^−1^)	0.01	4.04	0.34	0.77	0.00	1.37	0.30	0.34	0.02	1.84	0.30	0.41	0.01	2.61	0.43	0.67	0.00	2.19	0.25	0.40
Ni (μg L^−1^)	0.33	11.30	1.98	2.18	0.19	4.47	1.85	1.20	0.54	31.70	6.21	8.21	0.36	7.47	2.40	2.04	0.03	6.82	2.26	1.60
Cu (μg L^−1^)	0.26	326.00	23.52	62.96	0.38	68.00	10.89	14.09	0.73	53.00	8.58	12.50	0.21	47.50	11.11	14.70	0.30	46.20	5.78	9.50
Zn (μg L^−1^)	0.91	235.00	28.51	44.96	1.54	539.00	121.19	137.02	4.74	7820.00	696.78	1696.41	1.06	564.00	84.23	129.48	0.00	353.00	34.90	68.64
As (μg L^−1^)	0.26	5.46	1.40	0.98	0.06	3.88	1.01	0.91	0.19	2.75	1.22	0.80	0.14	2.57	1.06	0.74	0.51	4.74	2.03	1.18
Se (μg L^−1^)	0.00	0.45	0.03	0.11	0.00	0.50	0.03	0.10	0.00	4.73	0.78	1.39	0.00	0.56	0.03	0.12	0.00	1.09	0.06	0.20
Sr (μg L^−1^)	16.80	528.00	219.26	138.26	7.16	496.00	154.92	148.77	19.70	494.00	198.15	145.87	5.05	508.00	158.62	154.17	52.50	1190.00	305.14	205.76
Ag (μg L^−1^)	0.00	0.03	0.00	0.01	0.00	0.71	0.03	0.13	0.00	0.03	0.00	0.01	0.00	0.15	0.02	0.04	0.00	0.35	0.02	0.06
Cd (μg L^−1^)	0.00	0.54	0.05	0.10	0.00	0.88	0.10	0.16	0.00	1.45	0.30	0.38	0.00	0.91	0.17	0.25	0.00	0.79	0.08	0.16
Ba (μg L^−1^)	5.71	99.70	45.65	24.97	3.52	80.00	38.06	22.38	0.00	311.00	63.71	63.51	0.00	167.00	42.71	37.51	0.00	265.00	86.12	64.96
Tl (μg L^−1^)	0.00	0.02	0.00	0.00	0.00	0.10	0.01	0.02	0.00	0.08	0.02	0.02	0.00	0.13	0.02	0.03	0.00	0.08	0.01	0.02
Pb (μg L^−1^)	0.00	40.80	2.77	7.57	0.00	11.70	2.51	3.09	0.00	68.00	13.06	16.83	0.00	65.60	12.82	15.36	0.00	85.10	6.92	15.94
Na (mg L^−1^)	41.88	274.98	105.09	53.62	20.66	219.02	81.45	42.72	25.95	105.45	84.76	21.11	12.81	139.87	77.12	34.83	38.21	903.20	110.99	145.99
K (mg L^−1^)	12.43	240.70	31.79	39.69	3.12	40.60	18.31	9.56	7.08	29.75	20.45	7.46	3.90	31.25	18.15	9.31	8.84	196.50	31.46	33.68
Mg (mg L^−1^)	1.20	34.65	11.93	9.39	0.59	35.30	10.04	10.30	0.12	34.14	10.09	8.56	0.45	34.01	8.55	9.27	1.06	38.43	13.41	9.90
Ca (mg L^−1^)	4.68	62.44	31.67	14.87	0.40	61.76	21.98	18.57	1.70	66.46	34.49	15.73	3.92	60.31	25.99	17.59	7.39	96.31	45.48	17.38
Fe (mg L^−1^)	0.00	1.61	0.25	0.40	0.00	1.31	0.33	0.46	0.00	2.24	0.46	0.65	0.00	2.26	0.48	0.68	0.00	9.17	0.90	1.70

The content of trace metals in the studied rainwater runoff was much lower than the European average (Zawilski and Sakson 2013), with the exception of copper concentrations in sewer 1 and zinc in sewer 3 (Table 4). Copper is increasingly used in single-detached dwellings, hence the increased amount in sewer 1, while zinc often has a high concentration in the runoff from industrial areas. It is also sometimes used in glass production; hence, its increased amounts in runoff may possibly have originated from the glass factory.

Examined macroelements in most cases showed a much smaller concentration in the discharged storm water than in the water of the receiver. Rainwater runoff thus contributed to a slight reduction of their concentrations in the river water, but it was statistically significant only in the case of the largest discharge from collector no. 2 (Table 2).

Generally, the impact of storm water runoff on the water quality of the receiver was significantly different with respect to the Cybina River and the Antoninek Reservoir. Despite the large discharge of pollutant loads into the reservoir, statistically significant changes in particular variables of water quality were rarely observed (Table 2). This demonstrates the importance of the dilution process in a much larger volume of the reservoir than in the case of the river and accelerated sedimentation of the suspended matter with adsorbed pollutants compared to the river.

### Relationship between the elements and the environmental parameters

**Fig. 2:**
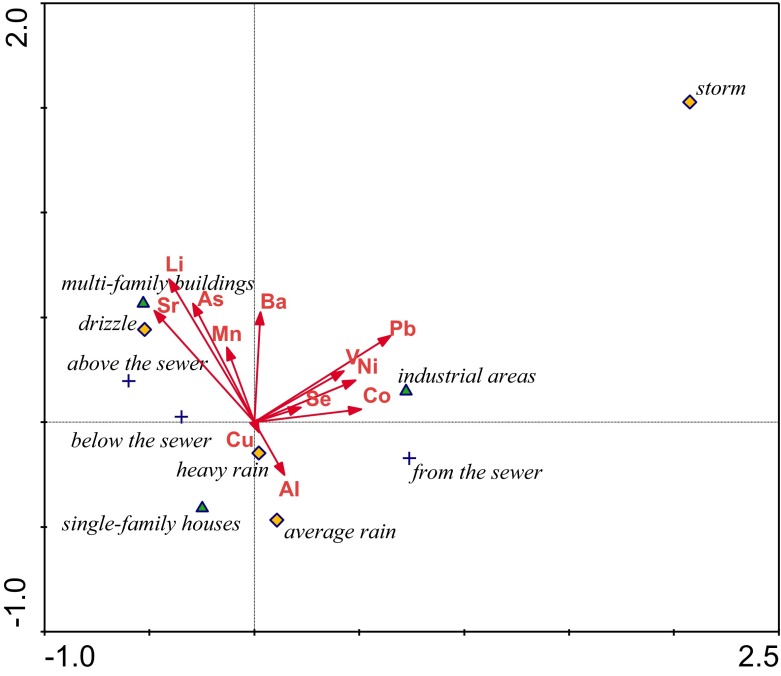
CVA model of relationship among land use, sampling point position, rain intensity, and analyzed trace elements (*p* < 0.05)

CVA proved that 12 elements, divided in the model into 3 groups (Fig. 2), were statistically important. The first of them were Sr, Li, As, Mn, and Ba, which were associated with the area of the multi-family settlement and small rainfall intensity. This group of metals is connected more with the river water (more above then below the sewer outlet) than with rainwater runoff. Que Hee (1994) stated that Mn is leached from the soil by acid rain. For industrial areas, the predictive variables were Pb, Ni, V, Co, and Se, but only during storm events and for the samples taken from the sewer. Lead was the most important in this group. According to Que Hee (1994) lead may leach to rain from the Pb-based paints, used for painting of industrial construction exteriors. It is also present in highway storm water runoff, as a product of car tire attrition (McKenzie et al. 2009). Because two of the industrial areas under study are related to car production and servicing and one of them also includes a section of motorway, this source is also very likely. Aluminum was the best explanatory variable for the samples of rainwater sewage from the residential area during rather intensive rain. This element can be easy diluted in such conditions. In this group, copper is also present, probably originating from the roofs of the residential houses (Göbel et al. 2007). The best predictive variables for the river waters, both upstream and downstream of the sewer, were elements from the first group, studied during drizzling rain. Using the backward elimination method, it was stated that the best explanatory variables for this model are Pb, which explains 22.2 % of variance, and Li, 16.7 %.

**Fig. 3:**
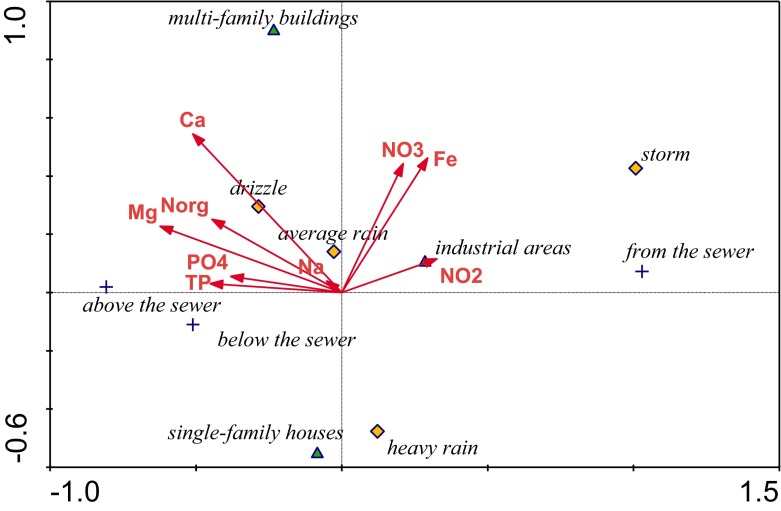
CVA model of relationship among land use, sampling point position, rain intensity, and analyzed macroelements as well as iron and forms of nitrogen and phosphorus (*p* < 0.05)

The model of the relationship of macroelements (together with iron and forms of N and P) (Fig. 3) and land use (together with rain intensity and sampling site) distinguished two groups of predictive variables. The first includes nitrates, nitrites, and iron, correlated with the runoff of heavy storms from industrial areas and the second, which includes Ca, Mg, N org., TP, and DRP, correlated with river water upstream and downstream of the sewer outlet during moderately intense rain events and partially dependent on the multi-family settlement. Leaching of these elements from soils was frequently reported (e.g., Watmough et al. 2005; Qin et al. 2013). Their correlation with river water, whose quality depends on the agricultural catchment, is thus reasonable. According to the sewer outlets, the relationship of these variables with storm waters from residential areas was rather expected. However, Brezonik and Stadelmann (2002) have stated a negative correlation for almost all constituents and size of suburban residential areas. Comparing the studied areas of multi-family and single-family houses, we observed a negative correlation of these variables with the population density. Larger areas covered by grass and other plants more efficiently trap constituents, compared to the urbanized areas. Densely populated areas are also subject to intensive low-chimney emission. Dust, soot, and debris falling from chimneys could be an additional source of these variables because they can easily leach from coal ash, as reported by Gentzis and Goodarzi (1999). This source is very probable, as coal combustion is still frequently used in for heating in Poland.

Interestingly, nitrates are correlated positively with the industrial land use (partly also with the multi-family settlement) and negatively with the residential areas. This nutrient can be easily released from agricultural areas; therefore, it was expected to be present in the outflow from the gardens located in the residential areas. Sarukkalige and Priddle (2011) obtained similar results in the industrial lands adjacent to the town of Victoria Park in Western Australia. In residential areas, vegetation prevents water erosion. Additionally, plants intensively absorb nitrates, so they are not flushed away even by heavy rains. The opposite is observed in industrial landscapes with abundant impermeable surfaces and high water erosion during storm events.

**Fig. 4:**
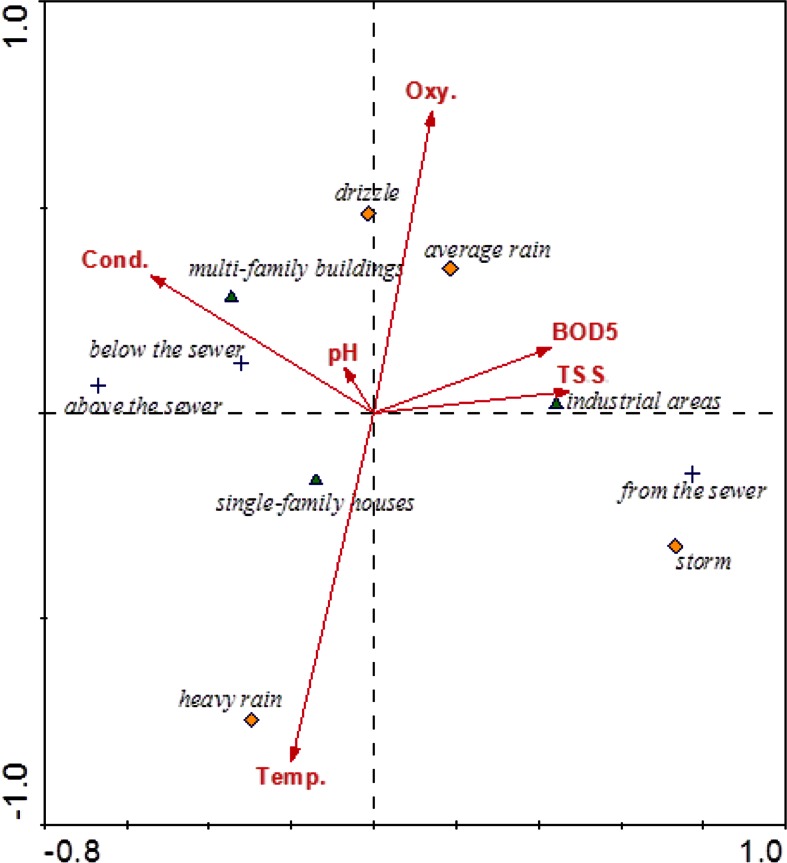
CVA model of relationship among land use, sampling point position, rain intensity, and such analyzed environmental variables as temperature (*Temp.*), conductivity (*Cond.*), total suspended solids (*TSS*), dissolved oxygen (*Oxy.*), and *BOD*_*5*_ (*p* < 0.05)

CVA models proved that land use is a very important variable, differentiating many elements and compounds observed in runoff. It confirms the earlier statement of Brezonik and Stadelmann (2002) with regard to the role of land use in the release of 10 common constituents. They stated (i.a.) that higher concentrations of TSS and organic matter flow out from the industrial areas than residential areas; this was confirmed in our case studies (Fig. 4). The model presented in Fig. 4 shows interdependencies between the water sampling points (upstream, downstream, and from the rainwater collector), location (industrial areas, residential areas, and blocks of flats), rain intensity, and environmental variables such as pH, conductivity, oxygen, BOD_5_, temperature, and TSS. BOD_5_ and TSS are closely dependent on each other and are present mainly in the outflow from industrial areas. This is due to a large share of impervious surfaces (51.6–76.0 %), which are easily flushed, and thus, the suspensions, especially organic, in large quantities are transported to the receiver. Such a dependence of the amount of washed out suspensions on the size of impervious areas was already stated by Deletic et al. (1997) and Crobeddu and Bennis (2007). Both BOD_5_ and suspended solids are dependent on very intensive rainfall (the storm), which is responsible for the flushing of organic matter particles. However, they are also dependent on the average rainfall (Fig. 4), which is of a frontal type and therefore lasts long enough to wash off impervious surfaces. This is because the kinetic energy responsible for particle erosion depends not only on the instantaneous rainfall intensity but also on time (Fornis et al. 2005). TSS are also responsible for the large presence of heavy metals in the runoff from industrial areas (Fig. 2), since a large amount of chemical elements are associated with particles (Crobeddu and Bennis 2007). The negative correlation of temperature and oxygen content is associated with decrease of the solubility of oxygen in rainwater with the increased temperature. Thus, higher oxygen content is related to the water discharged from the sewers during drizzle and average rain, as they belong to frontal rains, present mainly in cold seasons. Conductivity is mostly associated with the river waters, both at the station above and below the sewer outlet. It is negatively dependent on the rainwater discharged from the sewer systems. However, this negative relationship applies only to the storm water flushing the surface of the industrial catchment, not leaching the minerals from the soil (Göbel et al. 2007).

## Conclusions

1. Minimal values of pH from the list of non-priority pollutants were lower than 6.5 in storm water discharged by sewers from the area of car production and multi-family housing, which can have a chronic influence on aquatic organisms. Moreover, a decreasing trend was also observed for pH in the river under the influence of discharged storm water.
2. As many as 14 of the 22 analyzed elements in the runoff from the largest impervious area discharged from sewer no. 2 had a statistically significant impact on the river water quality. The greatest impact was observed in the case of cadmium, zinc, lead, and cobalt, but copper had the strongest toxic effect on aquatic organisms. Concentration of Cu in river water below all sewers exceeded acute values for some aquatic animals, e.g., cladocerans in 74 % of cases.
3. TSS in the rainwater runoff was high, resulting in a marked increase of its values in the river water. These suspensions easily sediment in the reservoir causing coprecipitation of adsorbed trace metals, which may cause a serious threat to the benthic invertebrates.
4. Contents of ions of elements in water depended on the location of the water sampling station. In rainwater collected from industrial and service areas, higher concentrations of Cd, Pb, Ni, Se, Tl, and Zn were found than in water collected from the area of a housing district with predominating multi-family houses.
5. Concentrations of macroelements in the river water to a greater degree depended on the catchment area than on the storm water runoff.
6. The reservoir situated in the river course had an important influence on the elimination of storm water pollution, despite its very short water residence time (0.5 day).
7. Since many variables and elements supplied to the receiver with rainwaters clearly affect the water quality, it is necessary to reduce the external loads and to determine acceptable impact, without affecting the native biodiversity negatively.

## Abbreviations

BOD: Biochemical oxygen demand
CCC: Criterion of continuous concentration
CMC: Criterion of maximum concentration
COD: Chemical oxygen demand
CVA: Canonical variation analysis
TSS: Total suspended solids

## References

[CR1] Brezonik PL, Stadelmann TH (2002). Analysis and predictive models of stormwater runoff volumes, loads, and pollutant concentration from watersheds in the Twin Cities metropolitan area, Minnesota, USA. Water Research.

[CR2] Brinkman SF, Johnston WD (2012). Acute toxicity of zinc to several aquatic species native to the Rocky Mountains. Archival Environental Contamination Toxicology.

[CR3] Brodie IM, Egodawalta P (2011). Relationships between rainfall intensity, duration and suspended particle washoff from an urban road surface. Hydroogyl Research.

[CR4] Brylińska M (1986). Freshwater fish of Poland.

[CR5] Budai P, Clement A (2007). Estimation of nutrient load from urban diffuse sources: experiments with runoff sampling at pilot catchments of Lake Balaton, Hungary. Water Science Technology.

[CR6] Burton A, Pitt R (2001). Stormwater effects handbook: a toolbox for watershed managers, scientists, and engineers.

[CR7] Clean Water Act (1972). Federal water pollution control act amendments of 1972. Public Law.

[CR8] Clearwater, S.J., Thompson, K.L., & Hickey, C.W. (2013). Acute toxicity of copper, zinc, and ammonia to larvae (Glochidia) of a native freshwater mussel *Echyridella menziesii* in New Zealand. *Archives of Environmental Contamination and Toxicology* in press 10.1007/s00244-013-9972-7 24292771

[CR9] Council Directive 91/271/EEC of 21 May (1991). concerning urban waste-water treatment. Official Journal L.

[CR10] Crobeddu E, Bennis S (2007). Washoff model of total suspended particles in an urban context [modele de lessivage des matières en suspension en milieu urbain]. Revue des Sciences de l’Eau.

[CR11] Deletic A, Maksimovic Č, Ivetic M (1997). Modelling of storm wash-off of suspended solids from impervious surfaces. Journal of Hydrauicl Research.

[CR12] Directive, W. F. (2000). Directive 2000/60/EC of the European parliament and of the council of 23 October 2000, establishing a framework for community action in the field of water policy. *Official Journal of the European Communities, L, 327*.

[CR13] Dougherty M, Dymond RL, Grizzard TJ, Godrej AN, Zipper CE, Randolph J (2006). Quantifying long-term NPS pollutant flux in an urbanizing watershed. Journal Environmental Engineering.

[CR14] Elbanowska H, Zerbe J, Siepak J (1999). Physico-chemical studies of water.

[CR15] EPA (1976). Quality criteria for water.

[CR16] EPA (1986). Quality criteria for water, 1986. U. S. Environmental Protection Agency, Report 440/5-86-001, Washington, D. C

[CR17] EPA (2007). Aquatic life ambient freshwater quality criteria—copper. 2007 Revision. CAS Registry No 7440-50-8, US EPA, Washington, DC.

[CR18] EPA (2009). National Recommended Water Quality Criteria. US EPA, Office of Water. http://www.epa.gov/ost/criteria/wqctable

[CR19] Fornis RL, Vermeulen HR, Nieuwenhuis JD (2005). Kinetic energy–rainfall intensity relationship for Central Cebu, Philippines for soil erosion studies. Journal Hydrology.

[CR20] Gentzis T, Goodarzi F (1999). Chemical fractionation of trace elements in coal and coal ash. Energy Source.

[CR21] Göbel P, Dierkes C, Coldewey WG (2007). Storm water runoff concentration matrix for urban areas. Journal Contaminant Hydrology.

[CR22] Gołdyn R (2000). Changes in biological and physico-chemical parameters of river water quality as a result of its damming in preliminary lowland reservoirs.

[CR23] Gołdyn, R., Dondajewska, R., Szeląg-Wasielewska, E., & Szyper, H. (2009). An appraisal of changes in seasonal water quality during passage through a shallow reservoir in Western Poland. *Environmental Monitoring Assessment, 151*, 181–188. 10.1007/s10661-008-0259-9 18437514

[CR24] Gołdyn R, Szeląg-Wasielewska E (2005). The effects of two shallow reservoirs on the phyto- and bacterioplankton of lowland river. Polish Journal Environmental Studies.

[CR25] ISO 17294–2 (2003) Water quality—application of inductively coupled plasma mass spectrometry (ICP-MS)—part 2: determination of 62 elements.

[CR26] ISO 7980 (1986) (E) Water quality. Determination of calcium and magnesium.

[CR27] ISO 9964–1 (1993) (E). Water quality. Determination of sodium and potassium.

[CR28] Johnson KA, Steinman AD, Keiper WD, Ruetz CR (2011). Biotic responses to low-concentration urban road runoff. Journal of the North American Benthological.

[CR29] Kasterka B, Kasterka B (1998). Badania zanieczyszczeń wód odpływających z wybranych zlewni gdańskiego systemu odwodnieniowego (GSO) [Studies on polluntants in waters drained from selected catchments of the Gdańsk Drainage System (GSO)].

[CR30] Ki SJ, Kang JH, Lee SW, Lee YS, Cho KH, An KG, Kim JH (2011). Advancing assessment and design of stormwater monitoring programs using a self-organizing map: characterization of trace metal concentration profiles in stormwater runoff. Water Research.

[CR31] Lis, J., & Pasieczna, A. (2005). Geochemical atlas of Poznań and surroundings. Soils, water sediments and surface waters. (in Polish)

[CR32] Marsalek J, Rochfort Q, Brownlee B, Mayer T, Servos M (1999). An exploratory study of urban runoff toxicity. Water Science Technology.

[CR33] McKenzie M, Hart P, Bai H, Jickling B (2009). Fields of green: restorying culture, environment, and education.

[CR34] Ministry of Environment Regulation (2006). On conditions to be met when introducing sewage to waters or to soil, and on substances particularly noxious to the aquatic environment. Dziennik Ustaw [The Journal of Law] 137, 984. (in Polish).

[CR35] Ministry of Environment Regulation (2011). On the classification of the status of surface waters and environmental quality standards for priority substances. Dziennik Ustaw [The Journal of Law] 257, 1545. (in Polish).

[CR36] Nocoń W, Barbusiński K, Nocoń K, Kernert J (2013). Changes in trace metal load in suspended solids carried along the river. Ochrona Srodowiska.

[CR37] Qin Z, Shober AL, Beeson RC, Wiese C (2013). Nutrient leaching from mixed-species Florida residential landscapes. Journal Environtal Quaility.

[CR38] Que Hee SS (1994). Availability of elements in leaded/unleaded automobile exhausts, a leaded paint, a soil, and some mixtures. Archives Environmental Contamination Toxicology.

[CR39] Robertson MJ, Scruton DA, Gregory RS, Clarke KD (2006). Effect of suspended sediment on freshwater fish and fish habitat. Canadian Technical Report Fisheries Aquatic Science.

[CR40] Rossi L, Chèvre N, Fankhauser R, Margot J, Curdy R, Babut M, Barry DA (2013). Sediment contamination assessment in urban areas based on total suspended solids. Water Research.

[CR41] Rzętała M, Jaguś A, Rzętała MA, Rahmonov O, Rahmonov M, Khak V (2013). Variations in the chemical composition of bottom deposits in anthropogenic lakes. Polish Journal Environmental Studies.

[CR42] Rzymski, P., Niedzielski, P., Klimaszyk, P., & Poniedziałek, B. (2014). Bioaccumulation of selected metals in bivalves (Unionidae) and *Phragmites australis* inhabiting a municipal water reservoir. *Environmental Monitoring Assessment*. doi:10.1007/s10661-013-3610-8. 10.1007/s10661-013-3610-8 PMC3969812 24407963

[CR43] Sarukkalige, R. & Priddle, S. (2011). Assessment of urban stormwater quality in Western Australia. In: Xuan, L (ed) *2nd International Conference on Environmental Engineering and Applications* (ICEEA 2011), Aug 19 2011, pp 52–56. Shanghai, China: International Association of Computer Science & Information Technology (IACSIT) Press

[CR44] Sawicka-Siarkiewicz H (2006). Wpływ wód opadowych odprowadzanych z powierzchni szczelnych na wody wodociągowe [The effect of precipitation waters from watertight surfaces on mains water]. Wodociągi Kanalizacja.

[CR45] Shirasuna H, Fukushima T, Matsushige K, Imai A, Ozaki N (2006). Runoff and loads of nutrients and heavy metals from an urbanized area. Water Science Technology.

[CR46] Taweel A, Shuhaimi-Othman M, Ahmad AK (2013). In vivo acute toxicity tests of some heavy metals to tilapia fish (Oreochromis niloticus). Journal Biology Science.

[CR47] Walna B, Kurzyca I (2009). Tendencies of changes in the chemical composition of precipitation in the Wielkopolski National Park. Journal of Water and Land Development.

[CR48] Watmough SA, Aherne J, Alewell C, Arp P, Bailey S, Clair T (2005). Sulphate, nitrogen and base cation budgets at 21 forested catchments in Canada, the United States and Europe (review). Environmental Monitor Assessment.

[CR49] Wetzel RG, Likens GE (2000). Limnological analyses.

[CR50] Zawilski M, Sakson G (2013). Assessment of total suspended solid emission discharged via storm sewerage system from urban areas. Ochrona Środowiska.

[CR51] Zębek E, Szwejkowska A (2014). Influence evaluation of pretreated storm water on analysis of cyanobacteria numbers in Jeziorak Maly urban lake at various precipitation rates. Ochrona Środowiska.

